# Development and Evaluation of Recombinant B-Cell Multi-Epitopes of PDHA1 and GAPDH as Subunit Vaccines against *Streptococcus iniae* Infection in Flounder (*Paralichthys olivaceus*)

**DOI:** 10.3390/vaccines11030624

**Published:** 2023-03-10

**Authors:** Xiuzhen Sheng, Honghua Zhang, Min Liu, Xiaoqian Tang, Jing Xing, Heng Chi, Wenbin Zhan

**Affiliations:** 1 Laboratory of Pathology and Immunology of Aquatic Animals, KLMME, Ocean University of China, Qingdao 266003, China; xzsheng@ouc.edu.cn (X.S.); zhh1628@stu.ouc.edu.cn (H.Z.); aquamedic@ouc.edu.cn (M.L.); tangxq@ouc.edu.cn (X.T.); xingjing@ouc.edu.cn (J.X.); chiheng@ouc.edu.cn (H.C.); 2 Function Laboratory for Marine Fisheries Science and Food Production Processes, Qingdao National Laboratory for Marine Science and Technology, Qingdao 266071, China

**Keywords:** *Streptococcus iniae*, multi-epitope vaccine, immune response, protective efficacy, flounder (*Paralichthys olivaceus*)

## Abstract

*Streptococcus iniae* is a severe Gram-positive pathogen that can infect a wide range of freshwater and marine fish species. In continuation of our earlier studies on the development of *S. iniae* vaccine candidates, pyruvate dehydrogenase E1 subunit alpha (PDHA1) and glyceraldehyde-3-phosphate dehydrogenase (GAPDH) were highly efficacious in protecting flounder (*Paralichthys olivaceus*) against *S. iniae*. In the present study, to investigate the potential of multi-epitope vaccination strategy to prevent flounder against *S. iniae* infection, the liner B-cell epitopes of PDHA1 and GAPDH proteins were predicted using a bioinformatics approach and were identified by immunoassay, and recombinant B-cell multi-epitopes of PDHA1 and GAPDH (rMEPIP and rMEPIG) containing immunodominant epitope-concentrated domains were expressed in *Escherichia coli* BL21 (DE3) and were used as a subunit vaccine to immunize healthy flounder, while recombinant PDHA1 (rPDHA1), GAPDH (rGAPDH) and formalin-inactivated *S. iniae* (FKC) served as controls. Then, the immunoprotection efficacy of rMEPIP and rMEPIG was evaluated by determining the percentages of CD4-1^+^, CD4-2^+^, CD8β^+^ T lymphocytes and surface-IgM-positive (sIgM^+^) lymphocytes in peripheral blood leucocytes (PBLs), spleen leucocytes (SPLs) and head kidney leucocytes (HKLs), as well as total IgM, specific IgM, and relative percentage survival (RPS) post immunization, respectively. It was found that fish immunized with rPDHA1, rGAPDH, rMEPIP, rMEPIG and FKC showed significant increases in sIgM^+^, CD4-1^+^, CD4-2^+^, and CD8β^+^ lymphocytes and production of total IgM and specific IgM against *S. iniae* or recombinant proteins rPDHA1 and rGAPDH, which indicated the activation of humoral and cellular immune responses after vaccination. Moreover, RPS rate of the multi-epitope vaccine rMEPIP and rMEPIG groups reached 74.07% and 77.78%, higher than that of rPDHA1 and rGAPDH (62.96% and 66.67%) and KFC (48.15%). These results demonstrated that B-cell multi-epitope protein vaccination, rMEPIP and rMEPIG, could give a better protective effect against *S. iniae* infection, which provided a promising strategy to design the efficient vaccine in teleost fish.

## 1. Introduction

*Streptococcus iniae* is a Gram-positive pathogen that is widely distributed in aquatic environments and has a wide host range, including fish and humans [1,2,3]. *S. iniae* has been isolated in Asia, the Americas and the Middle East, which has been reported to infect 22 species of wild or commercially farmed fish, including red sea bream (*Chrysophrys major*), flounder (*Paralichthys olivaceus*), turbot (*Scophthalmus maximus*) and tilapia (*Oreochromis nilotica*) [1,4], causing annual economic losses to the aquaculture industry estimated at over USD 100 million [5]. Flounder affected by *S. iniae* show typical signs, including hemorrhage, exophthalmia, abdominal distension, ascites, and lesions of the liver, kidney, spleen, and intestine [6], causing a large number of deaths and leading to great economic losses in aquaculture [4,5,7]. The use of antibiotics and chemicals can prevent outbreaks of *S. iniae* disease, mainly including carbendazim combined with erythromycin, β-lactams, penicillins, macrolides, and quinolones. In addition, baits supplemented with micro-ecological agents such as *Bacillus subtilis*, *Saccharomyces cerevisiae* and *Aspergillus oryzae* can improve the resistance of fish to *S. iniae* [2,8,9,10]. With the serious impact of *S. iniae* on fish cultures, using antibiotics is a challenge from ecological and economic points of view, and vaccine development against this pathogen has become an urgent need [11].

Fish vaccination can be considered as an effective immunity inducer and a standard protocol to prevent diseases in aquaculture [12]. However, it is reported that conventional vaccines are associated with some drawbacks, including weak immunity, multi-dose administration, allergenic potential and low safety [13]. For instance, a formalin-inactivated *Edwardsiella ictaluri* vaccine has a constrained capacity to enter fish [14]; inactivated vaccinations did not produce enough immunity against the red sea bream iridovirus or the salmon pancreas disease virus (SPDV) [15]. Therefore, new vaccines such as subunit vaccines, DNA vaccines, and multi-epitope vaccines are being highly researched. In many cases, subunit vaccines are less able to stimulate an effective immune response than killed or live whole-cell vaccine preparations. This is due to the limited number of components that stimulate the immune system and the lack of multiple antigens that represent whole-cell vaccines [14]. Because the genetic material must be relatively protected to enter the host cell, DNA vaccine construction methods and immunization methods are limited [14]. The epitope, including the conformational and linear epitope, is an antigenic determinant of the antigen molecule that is recognized by the immune system [16]. Epitope vaccines have the advantages of low toxicity, high immune targeting, and safety and stability due to the removal of non-immune relevant components and harmful parts of the antigen, and they can expand the width of the immune response through the combination of multiple epitopes, making them a popular topic in vaccine research [17,18]. A multi-epitope vaccine composed of a series of or overlapping peptides is therefore an ideal approach for the prevention and treatment of pathogen infections. Over the past few years, vaccines based on B and T-cell peptides/epitopes have successfully provided multi-factor benefits over conventional vaccines. In mammals, the B-cell epitope of the influenza virus surface glycoprotein hemagglutinin, fused with a heat labile enterotoxin B subunit (LTB) from *Escherichia coli*, forms an antigen that induces serum (IgA and IgG) and mucosal secretions (IgG), is involved in humoral immunity, promotes the expression of cytokines IL-1β, IL-8 and IL-4 in cellular immunity, and provides better immune protection against the pathogenic H1N1 swine influenza virus [19]. Recombinant epitope fusion proteins of linear B-cell epitopes of *Aeromonas hydrophila* outer membrane protein C (OmpC) and LTB from *E. coli* significantly elicit humoral and cellular immune responses in mice [20]. In teleost fish, the chimeric protein, formed by the fusion of the ribosomal P0 protein from *Lepeophtheirus salmonis* with the C-terminal of T-cell epitopes from tetanus toxin and measles virus, causes an increase in IgM antibody titers in tilapia (*Oreochromis niloticus*), North African catfish (*Clarias gariepinus*) and Atlantic salmon (*Salmo salar*) [21]. However, B-cell epitope-based vaccines in teleost fish have never been reported, and designing efficacious multi-epitope vaccines remains a great challenge.

Currently, subunit vaccines and DNA vaccines against *S. iniae* have been investigated [22,23,24,25,26]. Several proteins have been used as recombinant vaccine components to protect against *S. iniae* infection, including glyceraldehyde-3-phosphate dehydrogenase (GAPDH), Sip 11, MtsB, C5a peptidase (ScpI), enolase (ENO), fructose-bisphosphate aldolase (FBA), phosphoglucomutase (PGM), CAMP factor, and pyruvate dehydrogenase E1 subunit alpha (PDHA1) [6,22,23,27,28,29,30]. However, the vaccines are not yet commercially available. GAPDH, as a key enzyme in the glycolytic pathway, is a surface protein of *S. iniae*. It reversibly catalyzes the oxidative phosphorylation of glyceral-dehyde-3-phosphate into 1,3 bisphosphoglycerate and is associated with protein binding, cell signaling in many organisms, and immune evasion in bacteria [31,32,33], while PDHA1 is a phosphorylated protein involved not only in microbial biochemical metabolism but also in pathogenicity. For example, PDHA1 inhibitors have been reported to kill *E. coli* in vitro, and a synthetic inhibitor has been reported to kill both fungi and bacteria [34,35]. PDHA1 of *Mycoplasma mycoides* subsp., *Mycoplasma capricolum* subsp. and *Mycoplasma hyopneumonia* has been found to be an immunogenic protein [36,37,38]. In our previous study, PDHA1 and GAPDH of *S. iniae* have been confirmed to be protective antigens against *S. iniae* infection, and recombinant PDHA1 and GAPDH (rPDHA1 and rGAPDH) subunit vaccines are able to induce a specific antibody response and produce a higher rate of immune protection against *S. iniae* infection than inactivated *S. iniae* in flounder (*Paralichthys olivaceus*) [23]. Thus, PDHA1 and GAPDH are appropriate candidate antigens that can help in acquiring potential epitope-based vaccines.

In the present study, in order to provide a multi-epitope vaccine against *S. iniae* infection, the linear B-cell epitopes of GAPDH and PDHA1 were analyzed and identified by using in silico methods and immunoassay. Then, multi-epitope peptide proteins of PDHA1 and GAPDH (rMEPIP and rMEPIG) were synthesized and expressed in *E. coli* and were used as multi-epitope-based subunit vaccines to immunize the flounder. Subsequently, the protective efficacy against *S. iniae* in flounder was tested. Meanwhile, the humoral and cellular immune responses of flounder, including the production of total IgM and specific IgM, and the percentage of CD4-1^+^, CD4-2^+^, CD8β^+^, surface IgM positive (sIgM^+^) lymphocytes in peripheral blood leucocytes (PBLs), spleen leucocytes (SPLs) and head kidney leucocytes (HKLs), were investigated.

## 2. Materials and Methods

### 2.1. Ethics Statement

The usage of fish was in strict accordance with the recommendations of the Guidelines for the Use of Experimental Animals of Ocean University of China. The protocol for animal care and handling used in this study was approved by the Committee on the Ethics of Animal Experiments of Ocean University of China (permission number: 20190101). Before sacrificing and handling, experimental fish were anesthetized with 100 ng/mL ethyl 3-aminobenzoate methanesulfonic acid (MS222, Sigma, St. Louis, MO, USA), and all efforts were made to minimize suffering.

### 2.2. Fish, Bacteria and Antibodies

Healthy flounder (750 ± 50 g or 25 ± 5 g) were purchased from a fish farm in Rizhao, Shandong province of China, cultured in a continuous aerated and running seawater system at a temperature of 21 ± 1 °C, and confirmed *S. iniae* free by standard microscopical and bacteriological examination before experiment. The fish weighing 750 ± 50 g were used to produce flounder anti-*S. iniae* antibodies and flounder anti-recombined PDHA1 and GAPDH protein antibodies, while the fish weighing 25 ± 5 g were used for vaccination and challenge after acclimated to the laboratory environment for one week.

The pathogenic strain of *S. iniae* was stored in our laboratory. The strain was incubated in brain–heart infusion (BHI) medium at 28 °C for 24 h. The bacterial suspension was collected by centrifugation and resuspended in phosphate-buffered saline (PBS, pH = 7.2). Then, *S. iniae* was inactivated by using a final concentration of 2% formalin as described previously [39], and formalin-killed cells (FKC) at 1.0 × 10^8^ cfu/mL were used for immunization of flounder. Live *S. iniae* of 1.0 × 10^7^ cfu/mL was used for challenge experiment at 5 weeks post immunization.

The flounder anti-*S. iniae* antibodies were produced via intraperitoneal injection with the mixtures of FKC (1.0 × 10^8^ cfu/mL) and an equal volume of Freund’s complete adjuvant (FCA, Sigma). Booster immunization was performed by injecting the same amount of FKC mixed with incomplete Freund’s adjuvant (FIA, Sigma) at week 2 after the initial immunization. Similarly, the flounder anti-rPDHA1/rGAPDH antibodies were produced via intraperitoneal injection with the rPDHA1/rGAPDH (2.0 mg/mL) mixed with an equal volume of FCA and a booster immunization with rPDHA1/rGAPDH mixed with FIA at week 2 [23]. Subsequently, blood was collected from the caudal veins of the flounder at week 4 after vaccination and kept at 4 °C overnight, and then, the serum was obtained by centrifugation at 5000× *g* for 20 min at 4 °C and stored at −20 °C for later use. Furthermore, mouse monoclonal antibody (MAb) 2D8 against flounder serum IgM [40], rabbit polyclonal antibodies against flounder recombinant CD4-1, CD4-2 and CD8β that specifically recognized flounder T lymphocytes subsets, respectively [41], were previously produced by our laboratory.

### 2.3. Bioinformatics Analyses of B-Cell Epitopes of PDHA1 and GAPDH Proteins

The reference amino acid sequences of PDHA1 and GAPDH proteins were retrieved from NCBI (https://www.ncbi.nlm.nih.gov, accessed on 1 July 2019) in FASTA format with accession numbers of WP_003099247.1 and ACX85247.1, respectively. The B-cell epitopes were predicted by DNASTAR software and online prediction websites (IEDB: http://tools.iedb.org/main/bcell/, accessed on 1 July 2019; BCPREDS: http://www.cbs.dtu.dk/services/BepiPred/, accessed on 1 July 2019) according to the algorithms concerning the hydrophilicity (≥0), secondary structure (beta-turn or irregularly curled structure), flexibility, antigenic index (≥0) and surface probability (≥1). The 3D model of PDHA1 and GAPDH was generated using the SWISS MODEL server, and the selected B-cell epitopes were displayed. Linear synthetic peptides used in this study were synthesized by Genscript Biotech (Nanjing, Jiangsu, China) and purified to greater than 90% purity.

### 2.4. Identification of B-Cell Epitopes

Enzyme-linked immunosorbent assay (ELISA) was used for identification of B-cell epitopes. Wells of flat-bottom microplates (96-wells, Costar) were coated with 100 μL/well of synthetic polypeptide (50 μg/mL) for 3 h at 37 °C, and the flounder anti-*S. iniae* or anti-PDHA1/GAPDH serum (1:50 diluted in PBS) were added at 100 μL per well and incubated for 1 h at 37 °C. Non-immune flounder serum was used as a negative control. Following three washes, 100 μL anti-flounder serum IgM MAb 2D8 (1:1000 diluted in PBS) was added and incubated at 37 °C for 1 h. After washing, 100 μL goat-anti-mouse Ig-alkaline phosphatase conjugate (Sigma) diluted 1:5000 in PBS was added and incubated for 1 h at 37 °C. After the last three washes, 100 μL 0.1% (*w*/*v*) p-nitrophenyl phosphate (*p*NPP, Sigma) in 50 mM carbonate–bicarbonate buffer (pH = 9.8) containing 0.5 mM MgCl_2_ was added to each well and incubated at room temperature for 30 min in the dark. The reaction was stopped by adding 50 μL per well of 2 M NaOH, and absorbance was measured with an automatic ELISA reader (TECAN, Männedorf, Switzerland) at 405 nm. Each synthetic polypeptide was assessed in triplicate.

### 2.5. Design, Construction and Expression of the B-Cell Multi-Epitope Recombinant Antigens

The amino acid sequences of B-cell epitopes were analyzed through the different permutations and combinations of different peptide connectors (GGGG, EAAAK, AAY, KK, GPGPG), and the sequence of each epitope was selected to be relatively independent with good antigenicity parameters as the best tandem mode. Then, the chimeric gene was cloned into pET-28a (+) vector (Novagen, Beijing, China) and expressed in *E. coli* (DE3). Briefly, the constructed multi-epitope vaccine prokaryotic expression vector was inoculated in 6 mL of Luria–Bertani (LB) liquid medium (Kana^+^, Sangon, Shanghai, China) and incubated for 5 h at 37 °C with constant shaking. The culture was then expanded by transferring to 600 mL of LB liquid medium (Kana^+^, Sangon, Shanghai, China). When OD600 reached 0.6, 6 mL of Isopropyl β-d-Thiogalactoside (IPTG, Sangon, Shanghai, China) (0.1 M) was added, and the culture was continually shaken for 12 h. Induction was detected by SDS-PAGE. The recombinant multi-epitope peptides of PDHA1 (rMEPIP) and GAPDH (rMEPIG) were purified with His Trap Ni-NTA (GE Healthcare, Chicago, IL, USA) as described previously [23]. Purified recombinant peptides were complexed by dialysis solution with decreasing urea concentration (6M, 4M and 2M) and oxidized glutathione (0.12 g/L). The proteins were analyzed by SDS-PAGE and visualized with Coomassie brilliant blue R-250, and then verified using Western blot analysis with flounder anti-*S. iniae* serum [42]. The concentrations of rMEPIP and rMEPIG were determined using the Bradford method and were used as antigens for immunization experiments.

### 2.6. Fish Vaccination and Sampling

Flounder (25 ± 5 g) were equally divided into six groups (200 fish/group). For vaccination, fish were intraperitoneally injected with 100 µL purified rPDHA1, rGAPDH, rMEPIP, rMEPIG (2 mg/mL), and FKC (1.0 × 10^8^ cfu/mL) mixed with FCA (1:1). The control group was intraperitoneally injected with 100 µL of PBS and FCA (1:1).

Serum and leucocytes in the peripheral blood, spleen and head kidney of flounder were randomly sampled from fifteen fish in each group before immunization (0 d), and at 1 day (d), 3, 5, and 7 d, and at 2 weeks (w), 3, 4, 5, 6 and 7 w post immunization. For serum isolation, blood was collected from the caudal veins, prepared as described above, and then stored at −20 °C until use for ELISA assay. The leucocytes were obtained by Percoll density gradient centrifugation according to previously described procedures [23] and used for fluorescence-activated cell sorter (FACS) to analyze the percentage of CD4-1^+^, CD4-2^+^, and CD8β^+^ T lymphocytes and surface IgM positive (sIgM^+^) lymphocytes.

### 2.7. Flow Cytometric Immunofluorescence Analysis

The leucocytes (1.0 × 10^7^ cells/mL) were incubated with IgM MAb 2D8, rabbit polyclonal antibodies against CD4-1, CD4-2, and CD8β for 1.5 h at 37 °C. After washing three times with PBS containing 5% (*v*/*v*) newborn calf serum, the cells were incubated with FITC-conjugated goat-anti-mouse Ig (1:256 diluted in PBS, Sigma) for 45 min at 37 °C and washed again. The percentages of the CD4-1^+^, CD4-2^+^, CD8β^+^ T lymphocytes and sIgM^+^ lymphocytes in PBLs, SPLs and HKLs were analyzed with Accuri C6 cytometer (BD Accuri, Piscataway, NJ, USA). The myeloma culture supernatant instead of IgM MAb 2D8 and rabbit negative serum instead of polyclonal antibodies were used as negative controls.

### 2.8. Enzyme-Linked Immunosorbent Assay

ELISA was performed according to the procedure as described above. Specifically, for detecting the total antibody, wells of flat-bottom microplates (96-wells, Costar) were coated with 100 μL/well of previously stored flounder serum from each immune group at 4 °C overnight and then successively incubated with IgM MAb 2D8 as the primary antibody and alkaline phosphatase-conjugated goat-anti-mouse IgG as the secondary antibody. For detecting the specific antibody against *S. iniae* and recombinant proteins rPDHA1 and rGAPDH, 100 μL live *S. iniae* (1.0 × 10^9^ cfu/mL) or purified recombinant proteins rPDHA1 and rGAPDH (10 μg/mL) per well were coated and incubated with previously stored flounder serum (1:50) from each immune group as the primary antibody, IgM MAb 2D8 as the secondary antibody, and alkaline phosphatase-conjugated goat-anti-mouse IgG as the third antibody. The serum from the PBS group was used as control. Each serum sample was assessed in triplicate.

### 2.9. Challenge

At day 35 post-vaccination, fish were challenged by intraperitoneal injection of 100 µL live *S. iniae* (1.0 × 10^7^ cfu/mL). Afterward, mortalities were monitored over a period of 15 days after the challenge, and the relative percent survival (RPS) rates were calculated according to the equation: RPS = (1 − (% mortality in vaccinated fish/% mortality in control fish)) × 100%.

### 2.10. Statistical Analysis

The statistical analysis was performed using Statistical Product and Service Solution (SPSS) software (Version 20.0; SPSS, IBM, Armonk, NY, USA), differences between different groups were analyzed with one-way analysis of variance (ANOVA) and the results were expressed as mean ± SD. In all cases, the significance level was defined as *p* < 0.05.

## 3. Results

### 3.1. Prediction of B-Cell Antigenic Epitopes on PDHA1 and GAPDH Proteins

**Figure 1:**
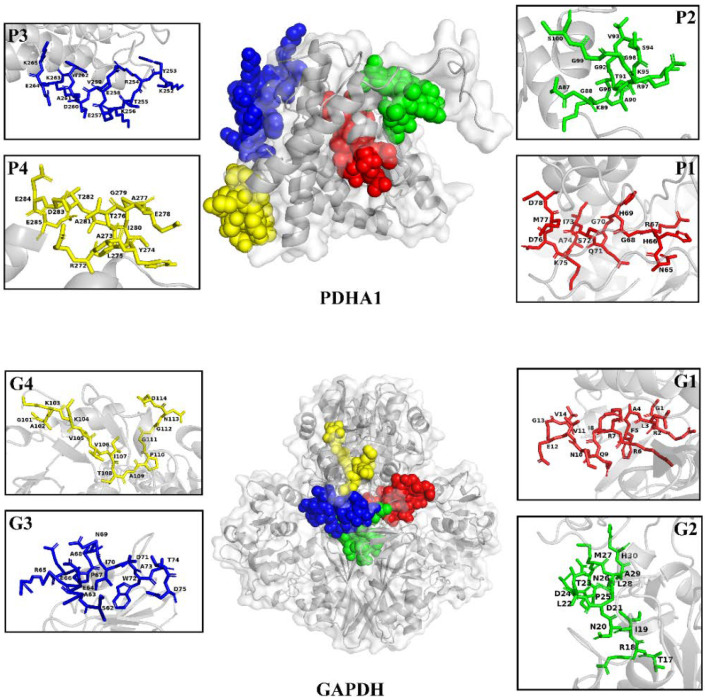
Three-dimensional structure of PDHA1 and GAPDH proteins and location of the predicted epitope peptides.

Four B-cell linear epitope-concentrated segments from PDHA1 (P1: 65-78aa; P2: 87-100aa; P3: 252-265aa; P4: 272-285aa) and four from GAPDH (G1: 1-14aa; G2: 17-30aa; G3: 62-75aa; G4: 101-114aa) exhibited higher scores for different parameters compared to other epitopes (Table 1). PDHA1 and GAPDH proteins showed a regular spatial structure, and the predicted peptide sequences of epitope P1 (red), P2 (green), P3 (blue) and P4 (yellow), as well as epitope G1 (red), G2 (green), G3 (blue) and G4 (yellow), were mostly located on the surface of the protein structure (Figure 1).

### 3.2. Antigenic Characteristic of B-Cell Epitopes

**Figure 2:**
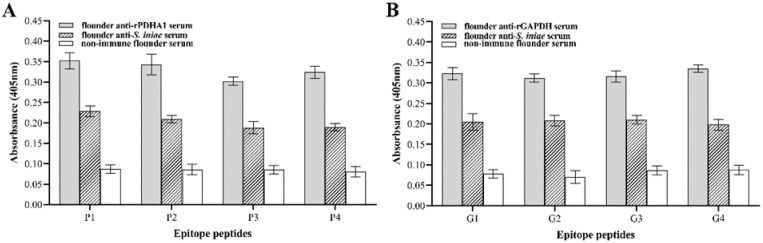
Reactions of the synthetic B-cell epitope peptides of PDHA1 and GAPDH to flounder serum by ELISA. (**A**) PDHA1 antigen epitope polypeptide and anti-PDHA1/anti-*S. iniae* serum; (**B**) GAPDH epitope polypeptide and anti-GAPDH/anti-*S. iniae* serum. Non-immune flounder serum was used as a negative control.

The ELISA results (Figure 2) showed that the epitope peptides P1, P2, P3 and P4 could all react with anti-PDHA1 serum, and the reaction activity was P1 > P2 > P4 > P3. Epitope peptides G1, G2, G3 and G4 could bind to anti-GAPDH serum antibodies of flounder, and the reaction activity was G4 > G1 > G3 > G2 in sequence. In addition, all epitopes could react with the anti-*S. iniae* serum of flounders, indicating that the selected epitopes retained the immune reactivity of PDHA1 and GAPDH proteins.

### 3.3. Production and Immunogenicity of Recombinant B-Cell Multi-Epitope Antigen Proteins

**Figure 3:**
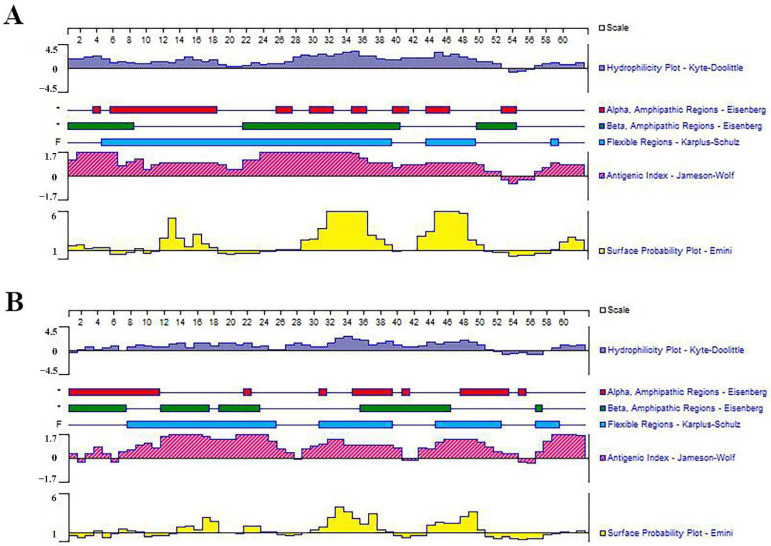
Prediction of hydrophilicity, antigenicity and surface probability of multi-epitope tandem. (**A**) MEPIP; (**B**) MEPIG.

**Figure 4:**
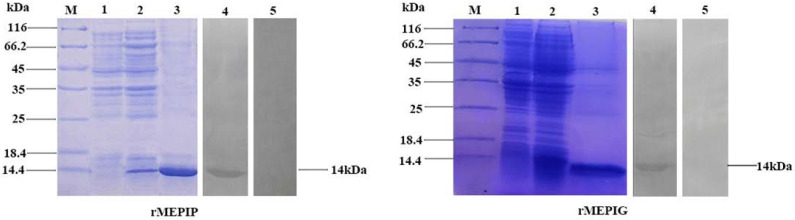
SDS-PAGE and Western blot analysis of the reactogenicity of rMEPIP and rMEPIG. Lane M: molecular mass marker; Lane 1: negative control without IPTG induction; Lane 2: *E. coli* induced with IPTG; Lane 3: purified recombinant protein; Lane 4: Western blot analysis using flounder anti-*S. iniae* serum; Lane 5: non-immune flounder serum was used as a negative control.

The optimized connection order of these mimotopes were confirmed to be P1-KK-P2-KK-P3-KK-P4 and G1-KK-G2-KK-G3-KK-G4, in which each epitope maintained its independence and antigenicity as analyzed by the Lasergene’s Protean program (Figure 3). SDS-PAGE analysis indicated that both the purified rMEPIP and rMEPIG showed a single band at 14 kDa, which was consistent with their theoretical molecular weight, suggesting that the recombinant proteins were efficiently purified. Western blot analysis indicated that the purified rMEPIP and rMEPIG proteins could be labeled well by flounder anti-*S. iniae* serum (Figure 4).

### 3.4. Variation of T Lymphocyte Subsets after Immunization

The PBLs, SPLs and HKLs were isolated from the vaccinated fish and were analyzed for forward scatter (FSC) and sideward scatter (SSC) patterns that represent the cell size and granularity, respectively. The dot plots of the gated lymphocytes and the fluoresce histograms of a peak levels of CD4-1^+^, CD4-2^+^ and CD8β^+^ lymphocytes in PBLs, SPLs and HKLs of flounder after immunization are shown in Figure S1.

**Figure 5:**
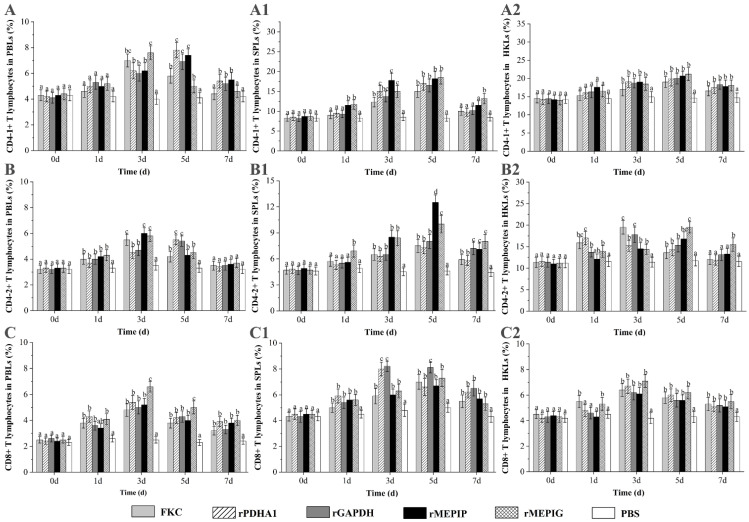
Variations of CD4-1^+^, CD4-2^+^ and CD8^+^ T lymphocyte percentages in PBLs, SPLs and HKLs of flounder after FKC, rPDHA1, rGAPDH, rMEPIP, rMEPIG and PBS immunization. (**A**,**A1**,**A2**): Variations of CD4-1^+^ T lymphocyte percentages in PBLs/SPLs/HKLs of flounder after immunization; (**B**,**B1**,**B2**): Variations of CD4-2^+^ T lymphocytes percentages in PBLs/SPLs/HKLs of flounder after immunization; (**C**,**C1**,**C2**): Variations of CD8β^+^ T lymphocytes percentages in PBLs/SPLs/HKLs of flounder after immunization. Values are shown as means ± SD of three fish. Different letters above the bar represent the statistical significance (*p* < 0.05) compared to each other at the same time point.

The percentages of CD4-1^+^ T lymphocytes in PBLs induced by FKC, rPDHA1, rGAPDH, rMEPIP and rMEPIG were significantly increased on the third day and were higher than those in PBS control (*p* < 0.05). The percentage of CD4-1^+^ T lymphocytes in PBLs reached a peak on day 3 in the FKC and rMEPIG groups (7.0% and 7.6%), and on day 5 in the rPDHA1, rGAPDH and rMEPIP groups (7.8%, 6.9% and 7.4%, respectively). The percentage of CD4-1^+^ T lymphocytes in SPLs began to rise on day 1 and reached a peak on day 5 in the rMEPIP (18.2%) and rMEPIG (18.5%) groups, which significantly increased on day 3 and peaked on day 5 in the FKC, rPDHA1 and rGAPDH groups (15%, 17% and 16.5%) (*p* < 0.05). Moreover, the percentages of CD4-1^+^ T lymphocytes in HKLs induced by FKC, rPDHA1, rGAPDH, rMEPIP and rMEPIG obviously rose on day 3, and reached a peak value on day 5, which were 19.0%, 19.9%, 20%, 20.7% and 21.2%, respectively, and then, they began to decline (*p* < 0.05) (Figure 5A).

The percentage of CD4-2^+^ T lymphocytes in PBLs was increased on day 1 as compared with the PBS group and before immunization (day 0), reached a peak of 5.5%, 6.0% and 5.8% in the KFC, rMEPIG and rMEPIP groups on day 3, and a peak of 5.5% and 5.4% in the rPDHA1 and rGAPDH groups on day 5, and then returned to the control level on day 7 (*p* < 0.05). In SPLs, the percentages of CD4-2^+^ T lymphocytes were obviously increased on day 3 in the FKC, rPDHA1, rGAPDH and rMEPIP groups and on day 1 in the rMEPIG group, and then peaked on day 5 in all groups (7.5%, 7.3%, 8.0%, 12.5% and 10.0%, respectively), but they were higher than control levels on day 7 in each group (*p* < 0.05). In HKLs, the percentage of CD4-2^+^ T lymphocytes increased on day 1, and reached a peak value of 17.0% on day 1 in the rPDHA1 group, a peak of 19.5% and 17.8% on day 3 in the FKC and rGAPDH groups, and a peak of 16.8% and 19.5% on day 5 in the rMEPIP and rMEPIG groups, respectively (*p* < 0.05) (Figure 5B).

The percentages of CD8β^+^ T lymphocytes in PBLs induced by FKC, rPDHA1, rGAPDH, rMEPIP and rMEPIG were significantly increased from the 1st to 3rd day, reaching a peak at 4.8%, 5.4%, 5.0%, 5.2% and 6.6%, respectively (*p* < 0.05). The percentage of CD8β^+^ T lymphocytes in SPLs increased on the 1st day in all immunized group, and peaked on the 3rd day in the rPDHA1 and rGAPDH groups (8.0% and 8.2%) and on the 5th day in the FKC, rMEPIP and rMEPIG groups (7.0%, 6.7% and 7.3%). Moreover, the percentages of CD8β^+^ T lymphocytes in HKLs were significantly increased on the 1st day and reached a peak on the 3rd day in the FKC and rMEPIG groups (6.4% and 7.1%), while they were increased and reached a peak on the 3rd day in the rMEPIP, rPDHA1 and rGAPDH groups (6.7%, 6.2% and 6.2%) (*p* < 0.05) (Figure 5C).

### 3.5. Variation of sIgM^+^ B Lymphocytes after Immunization

**Figure 6:**
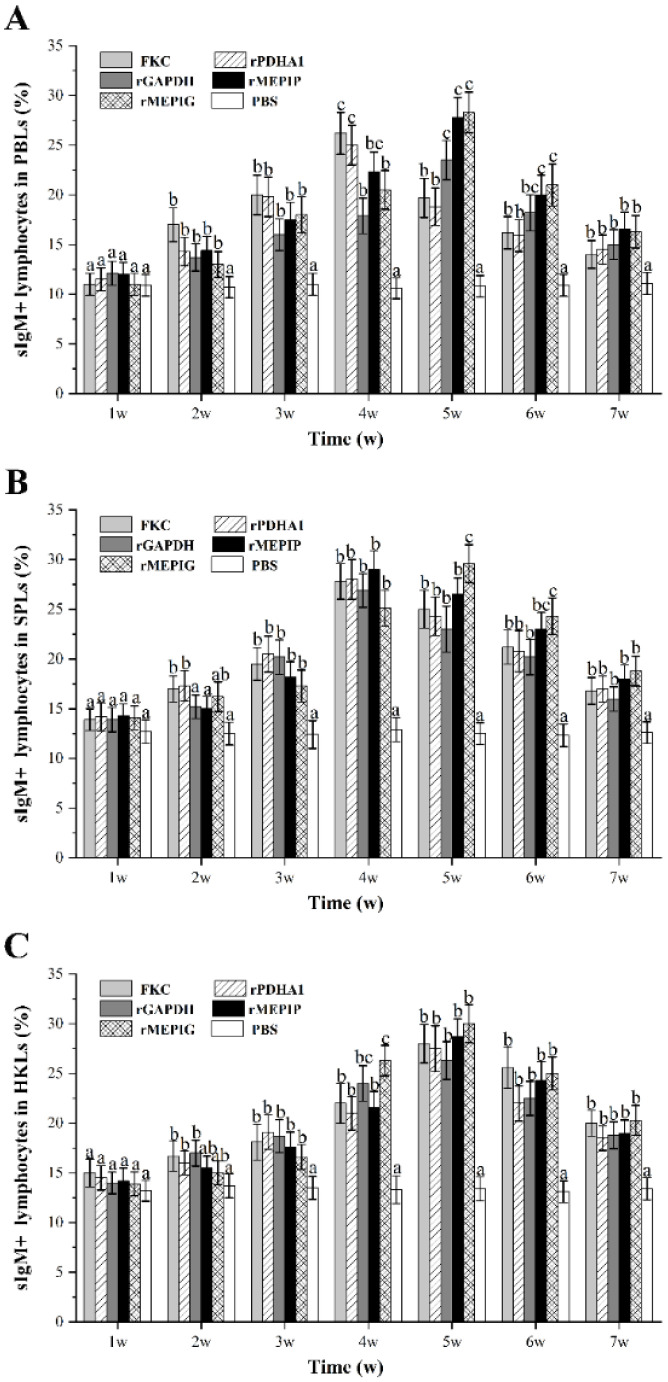
Variations of sIgM^+^ lymphocyte percentages in PBLs, SPLs and HKLs of flounder after FKC, rPDHA1, rGAPDH, rMEPIP, rMEPIG and PBS immunization. (**A**) Variations of sIgM^+^ lymphocytes in peripheral blood. (**B**) Variations of sIgM^+^ lymphocytes in spleen. (**C**) Variations of sIgM^+^ lymphocytes in head kidney. Values are shown as means ± SD of three fish. Different letters above the bar represent the statistical significance (*p* < 0.05) compared to each other at the same time point.

The gated lymphocytes in the FSC/SSC dot plot and representative fluorescence histograms at peak level after immunization are shown in Figure S2, and changes in percentages of sIgM^+^ B lymphocytes in different tissues of vaccinated fish are summarized in Figure 6. The levels of sIgM^+^ lymphocytes in PBLs induced in all groups began to rise from week 2 and were higher than those in the PBS group, peaked at week 4 or 5, and then began to decline. The percentage of sIgM^+^ lymphocytes in PBLs reached a peak at week 4 in the FKC and rPDHA1 groups (26.2% and 25.0%), and at week 5 in the rGAPDH, rMEPIP and rMEPIG groups (23.5%, 27.8% and 28.3%) (Figure 6A). The percentage of sIgM^+^ lymphocytes in SPLs began to rise at week 2 and reached a peak at week 4 in the FKC (27.8%) and rPDHA1 (28.0%) groups, which significantly increased at week 2 and peaked at week 5 in the rMEPIG group (29.6%) (*p* < 0.05). Moreover, the percentages of sIgM^+^ lymphocytes in SPLs induced by rGAPDH and rMEPIP reached a peak value at week 4, which were 26.9% and 29.0% (Figure 6B). In HKLs, the percentage of sIgM^+^ lymphocytes induced by FKC, rPDHA1, rGAPDH, rMEPIP, and rMEPIG groups all began to rise at week 2 and peaked at week 5, with peak levels of 28.0%, 27.5%, 26.3%, 28.7% and 30%, respectively (Figure 6C).

### 3.6. Detection of Total IgM and Specific IgM after Immunization

**Figure 7:**
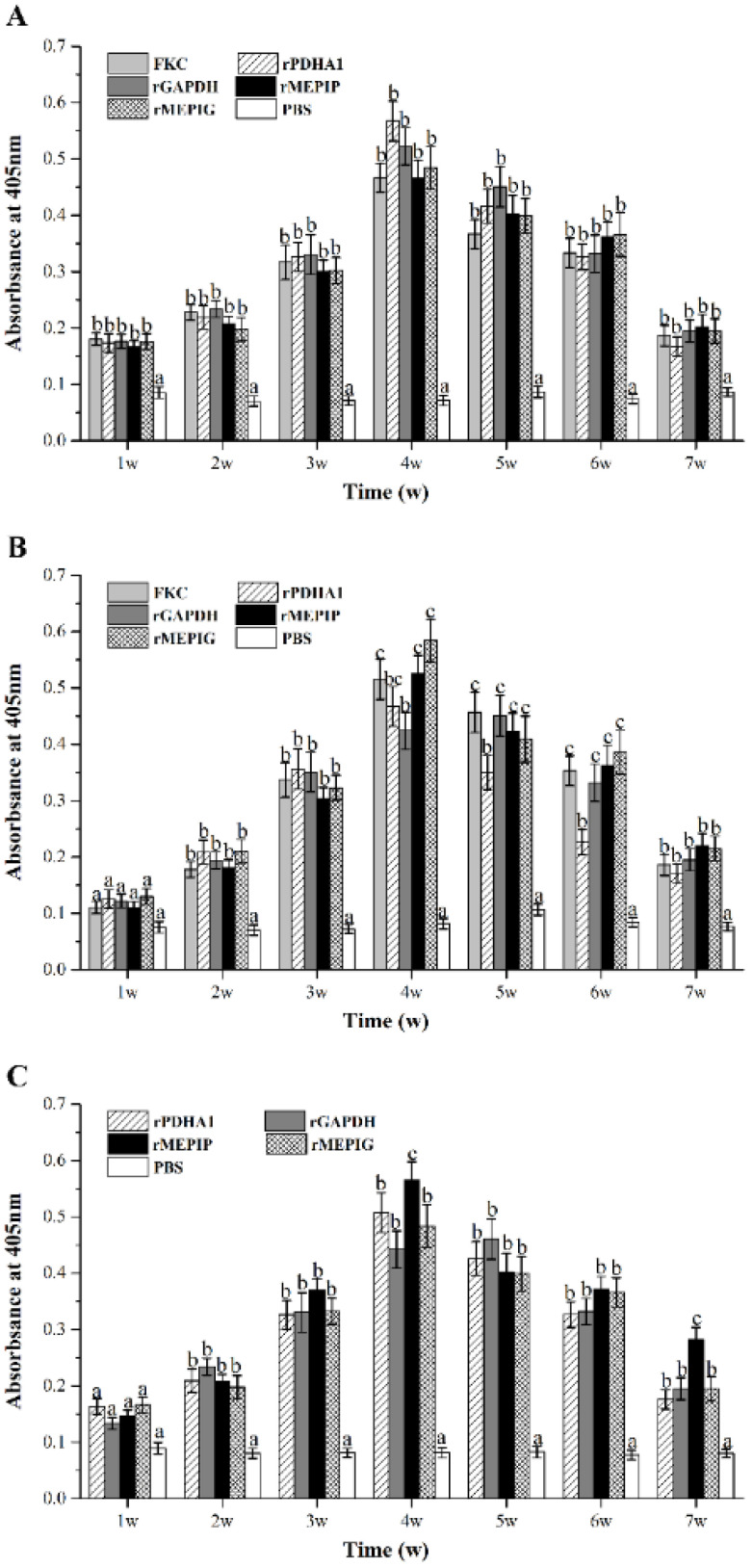
Variations of serum total IgM and specific IgM during 1–7 weeks after FKC, rPDHA1, rGAPDH, rMEPIP, rMEPIG, PBS immunization determined by ELISA. (**A**) Variations of serum total IgM. (**B**) Variations of specific IgM against *S. iniae*. (**C**) Variations of specific IgM against rPDHA1 and rGAPDH. Values are shown as means ± SEM of three fish. Different letters above the bar represent statistical differences (*p* < 0.05).

After immunization, flounder serum in the peripheral blood was taken at 1, 2, 3, 4, 5, 6 and 7 w, and ELISA was used to detect the changes of total and specific antibody levels in each group. The results showed that the total IgM level in peripheral blood of immunized flounder increased as compared with the PBS group, which began to rise at 1 w and reached a peak at 4 w, and then gradually decreased. The total antibody levels of rPDHA1 and rGAPDH immunized flounder were higher than those of other groups (Figure 7A).

For specific IgM against *S. iniae*, it presented a tendency to increase first and then decrease gradually in all vaccinated groups (Figure 7B), with a significantly higher level than the PBS control from week 2 after immunization (*p* < 0.05). The levels of specific anti-*S. iniae* IgM reached the maximum at week 5 in the rGAPDH group, and peaked at week 4 in the FKC, rPDHA1, rMEPIP and rMEPIG groups. In addition, the peak value induced by rMEPIP and rMEPIG was higher than that of the rPDHA1 and rGAPDH groups, but similar to that of FKC.

Furthermore, the level of specific IgM against recombinant proteins rPDHA1 and rGAPDH in all immunized groups was significantly higher than that of the PBS group from week 2 (*p* < 0.05) (Figure 7C), showing a trend of increase first and then decrease. In the rGAPDH group, it peaked at week 5 and then decreased slowly. In the rMEPIP, rMEPIG, and rPDHA1 groups, the level of anti-rPDHA1/rGAPDH-specific IgM peaked at week 4, with a higher level in the rMEPIP group.

### 3.7. Relative Percentage Survival

**Figure 8:**
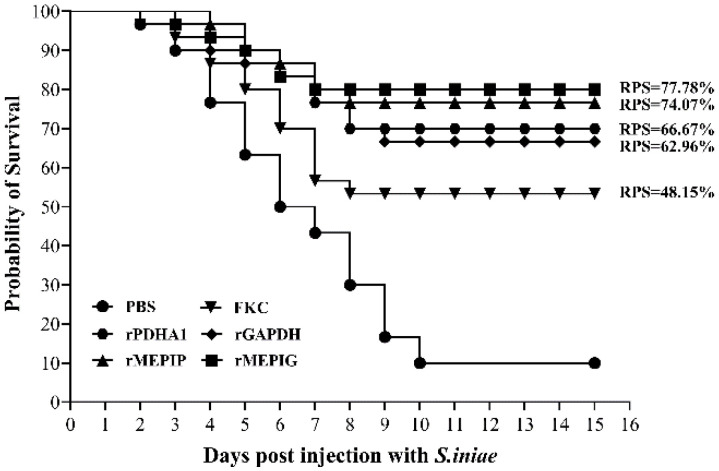
Cumulative mortality of flounder in FKC, rGAPDH, rPDHA1, rMEPIP, rMEPIG and PBS vaccination groups after challenge with *S. iniae*.

After *S. iniae* challenge, the infected fish showed typical signs of streptococcicosis, and the microbiological assay confirmed that *S. iniae* was the only bacterial pathogen re-isolated from the moribund fish (not shown). The accumulative mortality rate in the FKC, rGAPDH, rPDHA1, rMEPIP, rMEPIG, and PBS groups was 46.67%, 33.33%, 30%, 23.33%, 20% and 90%, corresponding to the calculated RPS of 48.15%, 62.96%, 66.67%, 74.07% and 77.78%, respectively (Figure 8). In addition, a significant analysis of mortality in each group by the Log Rank method revealed that mortality in the FKC, rGAPDH, rPDHA1, rMEPIP and rMEPIG groups was significantly different from that in the PBS group (*p* < 0.05) (Table 2).

## 4. Discussions

Epitopes are specific chemical groups in antigen molecules that determine antigen specificity, also known as antigenic determinant, which are the basic units of TCR/BCR and antibody specific binding, divided into T-cell epitopes and B-cell epitopes [16]. Epitopes, generally less than 20 amino acids in size, can be recognized by the body and stimulate the body to produce antibodies, which is the basis of protein antigenicity and the basic structure for inducing an immune response. In addition, epitopes have become a hot topic of research because of their great potential for vaccine design, disease prevention, diagnosis and therapy [43]. The accurate screening and identification of B-cell epitopes is a prerequisite for the study of epitope vaccines [44], and computerized bioinformatics software allows for rapid prediction of possible antigenic epitopes and reduces experimental effort [45]. The transmembrane topology of antigenic epitopes of MHC Class-1-Chain Related Protein-A was determined by TMHMM version 2.0, and the hydrophobicity of the protein sequence was analyzed using ExPASy ProtScale tool [46]. In addition to this, linear B-cell epitopes of nonstructural proteins of Enterovirus type 71 were predicted by ABCpred, BCPREDS and BepiPred online websites [47]. Unlike the bioinformatics methods above, we first used the BCRPEDS, IEDB online websites and DNASTAR software to analyze the amino acid sequences of PDHA1 and GAPDH as well as their secondary structure characteristics and physicochemical properties. Afterward, amino acid fragments with an antigenic index ≥ 0, surface probability ≥ 1 and hydrophilicity ≥ 0, located in beta-turn or irregularly curled structures, were selected. As a result, four possible B-cell linear epitopes of PDHA1 and GAPDH were screened. However, this method only focuses on the secondary structure of the amino acids and the physicochemical properties such as hydrophilicity and antigenicity, which may prevent the predicted antigenic epitopes from acting because they are buried inside the protein structure, thus reducing the accuracy of antigenic epitope prediction. In this study, the tertiary structures of PDHA1 and GAPDH were constructed by selecting amino acid sequences with >30% homology, and the selected antigenic epitopes were displayed on the 3D structure of the proteins to observe their position in the protein structure, which helped to predict the antigenic epitopes accurately. Our results showed that most of the four B-cell antigenic epitopes of PDHA1 and GAPDH were located on the surface of the protein 3D structure. By using flounder serum against *S. iniae* and PDHA1/GAPDH proteins at the optimal dilution of 1:50, and the synthetic B-cell epitope peptide proteins at the optimal concentration of 50 μg/mL, we confirmed that the selected B-cell epitope antigens had a good immune reactivity with anti-PDHA1/GAPDH and anti-*S. iniae* serum, and all eight epitope peptides presented a higher reactivity with anti-PDHA1/GAPDH than with anti-*S. iniae*, suggesting that the antigenic epitopes screened in this study could be used for further experiments, and further demonstrating that the combination of bioinformatics software and 3D structural position analysis could accurately predict valid B-cell antigenic epitopes.

Since epitopes have a small molecular weight and are easily degraded in vivo, multiple epitopes can be linked by genetic engineering to improve immunogenicity. It has been shown that when designing multi-epitope vaccines, suitable peptide junctions need to be introduced between adjacent epitopes to avoid the creation of new splice epitopes that may inhibit the immune response induced by the original epitopes [48]. In addition, the selection of suitable peptide junctions can improve the efficiency of protease hydrolysis and antigenic epitope delivery, enhancing the immune effect. The short peptide junction GGGS is linked to five B-cell epitopes of r5EPIS, which is purified and expressed by an *E. coli* expression system, and the constructed multi-epitope vaccine can induce an increase in the level of specific antibodies in the body with significant immune protection [49]. A multi-epitope vaccine was constructed by linking multi-epitope peptides of *Vibrio mimicus*’ outer membrane protein U with AAY junctions, and the multi-epitope vaccine was found to induce a higher level of immune response [50]. It was also found that different arrangements of amino acid sequences can affect the properties of proteins [51]. In this study, to select short peptide junctions to optimize the design of multi-epitope vaccines, the software DNAstar protean was used to analyze the linkage sequence and spacing of multiple epitopes. We found that the use of lysine–lysine (KK) as a linker, in the natural linking order of proteins, greatly reduced the creation of new spliced epitopes between adjacent epitopes, while the relative independence of the epitopes allowed for better induction of immune responses. KK is the cleavage site of cathepsin B, which facilitates in vivo cleavage of epitope peptides [52]. In addition, the short peptide junction is the preferred cleavage site for the proteasome, and the correct short peptide junction also improves the efficiency of intracellular proteasome cleavage of the epitope, thereby increasing the efficiency of antigen delivery. The *E. coli* expression system is by far the most widely used and most convenient prokaryotic expression system, but the *E. coli* expression system has the disadvantage that it is not conducive to peptide chain folding, allowing for misaggregation between some of the folded intermediate states, and resulting in some of the target proteins being in the form of inclusion bodies [53]. To solve this problem, this study focused on changing the expression conditions, including reducing the induction intensity (IPTG = 0.1 M), and then purifying the soluble fraction from the supernatant of the lysate in combination with His Trap Ni-NTA affinity chromatography, which also resulted in a sufficient soluble protein. In addition, the recombinant proteins were complexed by stepwise dialysis, with oxidized glutathione added as an oxidant at different stages of dialysis, and soluble recombinant proteins were obtained with high purity for subsequent experiments. The purified rMEPIP and rMEPIG were tested by Western blot with flounder anti-*S. iniae* antibodies, which showed significant specificity and provided a basis for subsequent validation of the vaccine.

In recent years, several studies have examined indicators other than RPS to evaluate vaccine effects, mainly including dynamic changes in lymphocyte ratios, antibody production and immune factor expression levels in fish after immunization [54,55,56,57,58]. Vaccines can induce changes in T and B lymphocyte ratios, and these changes are associated with specific immune protection [40,41,59]. In our laboratory, it was found that inactivated *Edwardsiella tarda* caused an increase in the ratio of T and B lymphocytes in the spleen and head kidney of flounder [58]. In addition, some studies in teleost fish have shown that CD4-1/-2 T lymphocytes and CD8 T lymphocytes are equivalent to helper and cytotoxic T lymphocytes in mammals, respectively [60,61,62,63]. In this experiment, flounder were immunized intraperitoneally with FKC, recombinant subunit vaccine rPDHA1 and rGAPDH, and multi-epitope vaccines rMEPIP and rMEPIG, we found that the percentage of CD4-1^+^ T lymphocytes induced by the rMEPIG group peaked on day 3 was higher than other groups, while the rMEPIP group peaked on day 5 in PBLs. In SPLs and HKLs, the percentage of CD4-1^+^ T lymphocytes induced by rMEPIP and rMEPIG all peaked on day 5 and were higher than other groups. For CD4-2^+^ T lymphocytes, the rMEPIP and rMEPIG groups peaked on day 3 and were higher than the other groups in PBLs, while both peaked on day 5 in SPLs and HKLs. For CD8β^+^ T lymphocytes, the rMEPIP and rMEPIG groups peaked on day 3 in PBLs and HKLs, and on day 5 in SPLs. These results indicated that multi-epitope vaccines rMEPIP and rMEPIG had good immunogenicity and could induce cellular immune responses involving T lymphocyte subsets. A study has shown that B-cell epitopes of avian leukosis virus subgroup J tandem-conjugated with short peptide junctions GAGS was used to construct a multi-epitope vaccine, which could induce humoral and cellular immune responses in chickens, which is consistent with our study [42]. Our results also indicated that the B-cell epitopes could be cascaded by peptide junctions to form new antigenic epitopes that entered the fish and stimulated the proliferation of T-cells in the same way as other antigens, causing a cellular immune response in the fish. Furthermore, the combination of T-cell epitopes and B-cell epitopes in an epitope vaccine can effectively stimulate cellular and humoral immune responses. It is reported that three T-cell epitopes and two B-cell epitopes of Urease B subunit (UreB) and *Helicobacter pylori* A (HpaA) are coupled using -KK-linkage and LTB as an adjuvant, eliciting cellular and humoral immune responses in mice [64]. Thus, our multi-epitope vaccines could also be designed to enhance the immune response by introducing T-cell epitopes and suitable vectors to make them more immunogenic in the future. Regarding the induction of B lymphocytes, this study found that the percentages of sIgM^+^ lymphocytes in PBLs and HKLs reached a peak at week 5 in the rMEPIP and rMEPIG groups, which were higher than the other groups. In SPLs, the percentage of sIgM^+^ lymphocytes reached a peak at week 4 in the rMEPIP group and at week 5 in the rMEPIG group. Moreover, the T lymphocytes reached peak levels earlier than the B lymphocytes, suggesting that the T-cell immune response occurred first after the multi-epitope vaccine and the recombinant protein vaccine immunized the fish. This was presumably due to activation of fish T lymphocytes and B lymphocytes, and then to regulation of B lymphocytes by T lymphocytes, but the specific mechanisms of activation and regulation need to be further investigated. In all tissues, the percentage of T lymphocytes in both the recombinant protein and FKC immune groups increased significantly from day 1 or 3, and the percentage of B lymphocytes increased significantly from week 3 compared to the PBS group, indicating that recombinant protein and FKC also elicited cellular and humoral immune responses in flounder. After entering the fish, the recombinant protein and FKC vaccine can be endocytosed by phagocytes and broken down by lysozymes into small peptides that bind to major histocompatibility complexes (MHCs) and that stimulate B lymphocytes through T-cells to produce antibodies [65,66]. The spleen and the head kidney are important immune organs of the fish, and the vaccine can stimulate the proliferation of lymphocytes in the spleen and the head kidney. In this study, the levels of CD4-1^+^/-2^+^ T lymphocytes in the peripheral blood were generally lower than those in the spleen and head kidney, and the levels of CD8β^+^ T lymphocytes and sIgM^+^ B lymphocytes were generally similar to those in the spleen and head kidney, which corresponded to the distribution of T/B lymphocytes in the peripheral blood and lymphoid tissues of the fish [41]. Fish batches, immune doses, protein concentrations, etc., might cause differences to varying degrees in lymphocyte levels in fish.

Vaccines cause the body to develop an immune memory, and the body rapidly produces antibodies with high titers and long maintenance at the next stimulation with the same antigen, making antibodies an important indicator of the protective effect of vaccines [67,68,69]. The detection of changes in serum antibody levels includes changes in total antibody levels, antibacterial-specific antibody levels, and anti-protein-specific antibody levels, which reflect the level of humoral immune response to antigens [70]. IgM is the primary antibody in fish and plays an important role in neutralizing pathogens through humoral immunity [71]. In this study, all vaccines induced a significant increase in total serum IgM levels from week 1 to 7 as compared with the PBS control, which peaked at week 4 with a higher peak level in the rPDHA1 and rGAPDH groups than the other groups, indicating that humoral immune responses were generated in flounder following antigenic immune stimulation. A multi-epitope vaccine, rSip-ClfA, which is constructed from B-cell epitopes of *Streptococcus agalactiae* Sip and *Staphylococcus aureus* ClfA proteins, can cause higher level of specific antibodies in mice than in the inactivated groups [72]. In this study, the levels of specific IgM against *S. iniae* and recombinant proteins rPDHA1 and rGAPDH in all immunized groups were significantly higher than that of the PBS group from week 2 to 7, and the FKC, rPDHA1, rMEPIP and rMEPIG groups all peaked at week 4, and the rGAPDH group peaked at week 5. In addition, the peak values of specific IgM antibody in the rMEPIP and rMEPIG groups were higher than those of the corresponding recombinant protein groups. Comparing the total antibody with the anti-*S. iniae* specific antibody levels, rMEPIP and rMEPIG induced a similar total serum antibody level to rPDHA1 and rGAPDH but a higher specific antibody level than the rPDHA1 and rGAPDH groups. Total antibody levels represent the amount of total serum IgM, whereas specific antibody levels are the amount of IgM secreted by B lymphocytes stimulated by antigenic epitopes. Multi-epitope vaccines are composed of B-cell epitope junctions that stimulate the host’s immune system to produce specific antibodies against pathogenic bacteria. rMEPIP and rMEPIG therefore elicited high levels of antibacterial-specific antibodies, although total antibody levels were slightly lower than that of the recombinant proteins in this study. In addition, these results were consistent with the relative percent survival, suggesting a correlation between the level of antibacterial-specific antibodies and the relative percent survival rate. Thus, the level of specific antibodies in this study was more indicative of the effectiveness of the vaccine.

The recombinant proteins PDHA1 and GAPDH have been shown to provide 75% and 62.5% relative percent survival, as well as higher sIgM^+^ lymphocyte ratios and antibody levels in our previous study [23]. On this basis, the designed and expressed multi-epitope vaccines rMEPIP and rMEPIG provided high relative percent survival rates of 74.07% and 77.78%, respectively. Compared to those provided by rPDHA1 (66.67%), rGAPDH (62.96%) and FKC (48.15%), the recombinant multi-epitope vaccines provided better immune protection against *S. iniae*. The differences in RPS of rPDHA1 and rGAPDH between the previous study and this one might be due to differences in the batch condition of the fish and the doses and concentrations used for immunization, and therefore, multiple doses and concentration groups could be used in subsequent experiments to fully analyze the effectiveness of the vaccine.

## 5. Conclusions

Based on our previous study, here, the antigenic epitopes of PDHA1 and GAPDH were analyzed, and eight B-cell linear epitopes were predicted. Recombinant B-cell multi-epitope vaccines rMEPIP and rMEPIG, sequentially linked by KK polypeptide junctions, were identified as immunogenic and were used for the immunization of flounder. The relative percent survival rates of rMEPIP and rMEPIG were 74.07% and 77.78%, which were higher than those of the rPDHA1 (66.67%), rGAPDH (62.96%) and FKC groups (48.15%). Moreover, the rMEPIP, rMEPIG, rPDHA1, rGAPDH and FKC groups all induced an increase in the levels of CD4-1^+^, CD4-2^+^, CD8β^+^ T lymphocytes and sIgM^+^ B lymphocytes, and the B-cell multi-epitopes vaccine groups obtained higher sIgM^+^ B lymphocyte levels and antibacterial specific antibodies than those in rPDHA1 and rGAPDH. All these results demonstrated that B-cell multi-epitope protein vaccination, rMEPIP and rMEPIG, could activate humoral and cellular immune responses and give a better protective effect against *S. iniae* infection, which provides a promising strategy to design an efficient vaccine in teleost fish.

## Figures and Tables

**Table 1:** The results of epitope prediction.

Protein	No.	Amino Acid Sequence and Position	Mean Antigen Index
Pyruvate dehydrogenase E1 subunit alpha (PDHA1)	P1	NHRGHGQSIAKDMD (aa65-78)	1.85
	P2	AGKATGVSKGRGGS (aa87-100)	2.48
	P3	KYRTKEEVDAWKEK (aa252-265)	2.01
	P4	RAYLTAEGIATDEE (aa 272-285)	1.02
Glyceraldehyde-3-phospate dehydrogenase (GAPDH)	G1	GRLAFRRIQNVEGV (aa1-14)	1.73
	G2	TRINDLTDPNMLAH (aa 17-30)	1.20
	G3	SAEREPANIDWATD (aa62-75)	1.96
	G4	GAKKVVITAPGGND (aa101-114)	1.48

**Table 2:** The results of the Log Rank method.

Group	Significance					
	PBS	FKC	rPDHA1	rGAPDH	rMEPIP	rMEPIG
PBS		0.0024 *	0.0001 *	0.0001 *	0.0001 *	0.0001 *
FKC	0.0024 *		0.1623 ^ns^	0.2519 ^ns^	0.0533 ^ns^	0.0360 *
rPDHA1	0.0001 *	0.1623 ^ns^			0.5697 ^ns^	
rGAPDH	0.0001 *	0.2519 ^ns^				0.2888 ^ns^
rMEPIP	0.0001 *	0.0533 ^ns^	0.5697 ^ns^			
rMEPIG	0.0001 *	0.0360 *		0.2888 ^ns^		

“*” indicates that the difference was statistically significant (*p* < 0.05) and “^ns^” indicates that the difference was not statistically significant.

## Data Availability

The data presented in this study are available upon request from the corresponding author.

## Supplementary Materials

The following supporting information can be downloaded at: https://www.mdpi.com/article/10.3390/vaccines11030624/s1, Figure S1. Flow cytometric analysis of T lymphocyte subsets labeled with rabbit polyclonal antibodies against CD4-1, CD4-2 and CD8β in PBLs, SPLs and HKLs. Figure S2. Flow cytometric analysis of IgM+ lymphocytes labeled with anti-serum IgM MAb 2D8.
