# Supplementary material for: Development and Evaluation of Recombinant B-Cell Multi-Epitopes of PDHA1 and GAPDH as Subunit Vaccines against Streptococcus iniae Infection in Flounder (Paralichthys olivaceus)
Source: Vaccines (Basel). 2023 Mar 10;11(3):624. doi: 10.3390/vaccines11030624 (PMC10051852; doi:10.3390/vaccines11030624)

Figure S1. Flow cytometric analysis of T lymphocyte subsets labeled with rabbit polyclonal antibodies against CD4-1, CD4-2 and CD8β in PBLs, SPLs and HKLs.

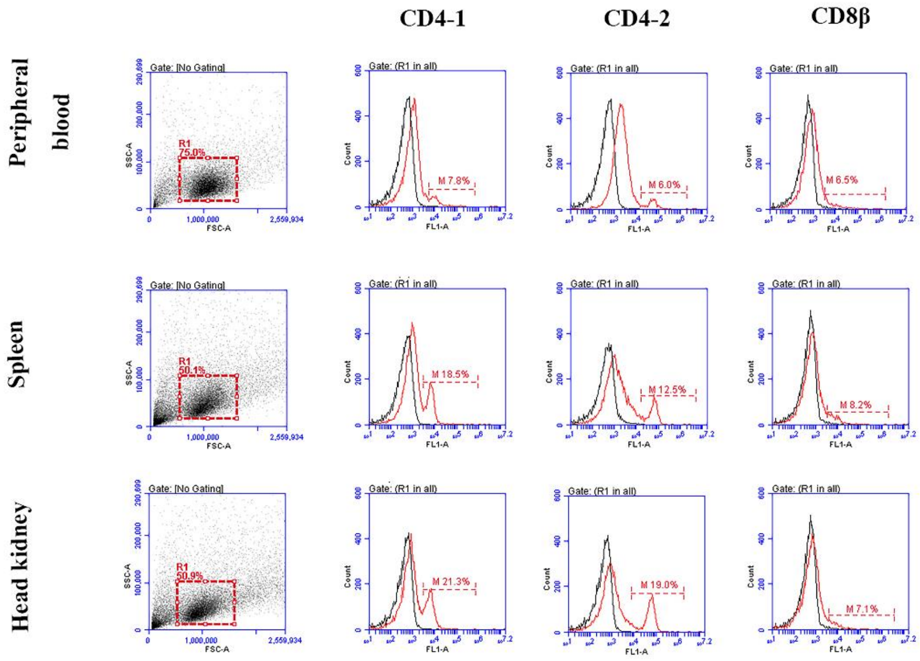

Figure S2. Flow cytometric analysis of IgM+ lymphocytes labeled with anti-serum IgM MAb 2D8.

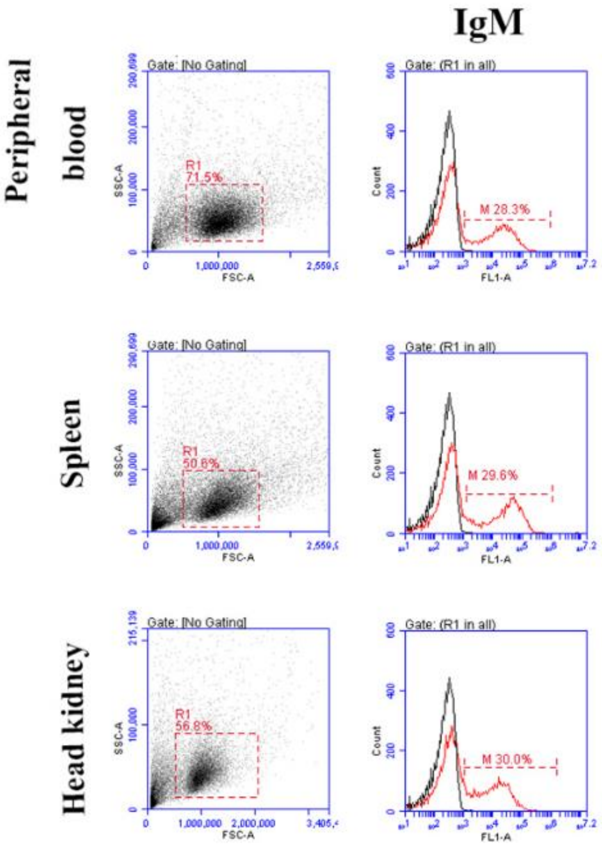

Supplement: Supplementary file 1 [file vaccines-11-00624-s001.zip › vaccines-2206486-supplementary.pdf]
